# Hepatitis B surface antigen impairs TLR4 signaling by upregulating A20 expression in monocytes

**DOI:** 10.1128/spectrum.00909-24

**Published:** 2024-09-09

**Authors:** Cong Wang, Chenlu Huang, Yaming Li, Jinjin Bai, Kuangjie Zhao, Zhong Fang, Jieliang Chen

**Affiliations:** 1 Shanghai Public Health Clinical Center, Fudan University, Shanghai, China; 2 Liver Cancer Institute of Zhongshan Hospital and Key Laboratory of Carcinogenesis and Cancer Invasion (Ministry of Education), Fudan University, Shanghai, China; 3 Key Laboratory of Medical Molecular Virology (MOE/NHC/CAMS), Research Unit of Cure of Chronic Hepatitis B Virus Infection(CAMS, Shanghai Frontiers Science Center of Pathogenic Microbes and Infection, School of Basic Medical Sciences, Shanghai Medical College, Fudan University, Shanghai, China; State Key Laboratory of Virology, Wuhan University, Wuhan, China

**Keywords:** hepatitis B virus, hepatitis B surface antigen, monocyte, TLR4, A20

## Abstract

Toll-like receptors (TLRs) play a crucial role in eliminating viral infection. Conversely, viruses have evolved various strategies to disrupt TLR signaling during chronic infection. In the case of hepatitis B virus (HBV), we previously reported that plasma hepatitis B surface antigen (HBsAg) is closely associated with impaired TLR responses in peripheral blood mononuclear cells from chronic hepatitis B (CHB) patients, but the reasons remain unclear. In this study, we investigated the mechanism by which HBsAg suppresses TLR4 signaling in monocyte cell lines. The monocyte cell line THP-1 was pretreated with HBsAg, followed by lipopolysaccharide (LPS) stimulation. Levels of proinflammatory cytokines and the activation of NF-κB, c-JNK, and ERK were examined. We found that HBsAg did not influence the LPS-induced activation of p65, but it disrupted NF-κB promoter activity through the ectopic expression of myeloid differentiation factor 88 (MyD88) and TAK1, suggesting that HBsAg can block downstream TLR4 signaling. Furthermore, we proved that LPS-induced polyubiquitination of tumor necrosis factor receptor-associated factor 6 (TRAF6) and the formation of the TRAF6-TAB2 complex were inhibited in HBsAg-pretreated cells. Interestingly, HBsAg led to a significant upregulation of A20, a ubiquitin-editing enzyme. Correspondingly, downregulation of A20 using siRNA restored LPS-mediated cytokines production, reflecting its crucial role in HBsAg-mediated inhibition of TLR4 signaling. These results demonstrated a novel mechanism by which HBsAg disrupts TLR4 signaling through the upregulation of A20, suggesting that targeting A20 may be a potential strategy to help restore monocyte functions.

**IMPORTANCE:**

Clearance HBsAg indicates a functional cure of HBV infection, but in chronic hepatitis B (CHB), it is hard to achieve. HBsAg has been found to regulate anti-viral immune responses, such as the activation of TLR. Our previous jobs proved that HBsAg negatively correlates with TLR2/4 activation in monocytes from CHB patients and blocks TLR2 ligand-indcuced IL-12 production in monocytes. However, how TLR4 signaling is affected by HBsAg remains unknown. In this study, we not only observed impaired TLR4 activation after pretreated monocytes with HBsAg but also identified HBsAg-induced A20 play a role in this impairment, which suggests that targeting A20 may be a viable strategy to restore monocyte functions in CHB.

## INTRODUCTION

Hepatitis B virus (HBV) is an enveloped DNA virus with clearance rates exceeding 95% in acute infections among adults. However, nearly 90% of newborns fail to resolve the initial infection, progressing to chronic infection, which can result in liver cirrhosis or hepatocellular carcinoma (HCC) (1, 2). Clearance of HBsAg indicates the functional cue of HBV infection, but it is hard to acquire immune responses against HBsAg in chronic infected patients.

Toll-like receptors (TLR), a family of pattern recognition receptors, are crucial for detecting invading pathogens and serve as a vital bridge between innate and adaptive immunity. Concurrently, viruses have been found to develop strategies to counteract TLR signaling transduction (3). TLR-induced antiviral responses are reported to directly inhibit HBV replication (4), whereas other studies suggest that HBV can counteract these TLR-mediated responses. HBsAg could effectively inhibit IFN-alpha production by TLR9-stimulated plasmacytoid dendritic cells (5), and HBV genome is able to trigger TLR3-dependent interferon responses (6). Our previous work also observed that the production of proinflammatory cytokines in peripheral blood mononuclear cells (PBMCs) from chronic HBV patients following challenge with TLR2 and TLR4 ligands was inhibited, which closely correlated with the concentration of plasma HBsAg (7). We further proved that TLR1/2 ligand-induced IL-12 generation was blocked by HBsAg in monocytes/macrophages by interfering with JNK (8). However, whether and how TLR4 signaling is regulated by HBsAg still remains unknown.

To clarify this, we investigated the impact of HBsAg on cytokines production in human monocytes stimulated with lipopolysaccharide (LPS), a canonical ligand of TLR4. Subsequently, we identified the specific step(s) within the TLR4 signal transduction pathway that was affected. The activation of NF-κB and MAPKs, the polyubiquitination of TRAF6, and the formation of the TRAF6-TAB2 complex were also assessed. Furthermore, we detected the expression levels of known negative regulators of TLR signaling in HBsAg-treated cells and observed an upregulation of A20. Transfection with A20-targeted siRNA could restore LPS-induced cytokines. Taken together, this study revealed a mechanism by which HBsAg inhibits monocytes and suggested that targeting A20 might benefit the treatment of hepatitis B.

## MATERIALS AND METHODS

### Cell culture

The human monocyte cell lines THP-1 and U937, obtained from Cell Bank of Chinese Academy of Sciences, were cultured in suspension culture in Roswell Park Memorial Institute (RPMI) 1640 medium (HyClone) with 10% fetal bovine serum (FBS; GIBCO), penicillin (100 IU/mL; GIBCO), and streptomycin (100 µg/mL; GIBCO), at 37°C in a 5% CO_2_ incubator. PBMCs from healthy donors, sourced from the Shanghai Blood Center, were isolated and cultured according to a previously described protocol (7).

### Hepatitis B surface antigen

Plasma HBV surface antigens (HBsAg) were purified from the pooled sera of HBV patients (Kehua Bio-engineering Co., Ltd., China) and identified using 10% SDS-PAGE. The presence of the native p24 and glycosylated gp27 forms of the S protein was confirmed by immunoblotting and silver staining (Fig. S1). The LPS contamination in the HBsAg preparation was below 100 pg of endotoxin per mg of HBsAg, as estimated by the limulus amebocyte lysate assay.

### RNA isolation and real-time PCR

Total cellular RNA was extracted with TRIzol reagent (Invitrogen). RNA was treated with DNase I (Takara) to eliminate genomic DNA, followed by reverse transcription using the Toyobo Ace reverse transcriptase, as per the manufacturer’s protocol. The cDNA samples underwent real-time PCR with primers specific to the genes enumerated in Table S1. For comparative analysis, the expression levels of the genes were normalized against GAPDH.

### Plasmids and transfection

The myc-MyD88 plasmid was constructed as previously described (9). For the construction of Flag-p65, p65 cDNA was amplified from THP-1 cell-derived RNA and inserted into the EcoR I/Not I sites of the pcDNA3.1 vector, which already contained three tandem Flag epitopes. The primers used were: forward, 5′-CCGGAATTCATGGACGAACTGTTCCCCCT-3′ and reverse, 5’- ATAAGAATGCGGCCGCTTAGGAGCTGATCTGAC-3′. The HA-TAK1 plasmid was kindly provided by Dr. Jun Ninomiya-Tsuji (North Carolina State University, US). Plasmids encoding siRNA against A20 (pU6-A20i) and or Renilla luciferase (pU6-Ctrli, as a control) were generously provided by Prof. Shoji Yamaoka (Tokyo Medical and Dental University, Japan). Plasmid transfections were conducted by electroporation using the Lonza/Amaxa nucleofector system according to the manufacturer’s instructions.

### Luciferase reporter gene assay

THP-1 cells (1 × 10^6^) were co-transfected with 100 ng of pNF-κB-Luc, 25 ng of pRL-TK (expressing Renilla luciferase; Promega), and 800 ng of MyD88, TAK1, or p65 expression plasmids. The cells were mock-treated or treated with 10 µg/mL HBsAg 2 h post-transfection for an additional 24 h. The cells were then lysed with 1 × passive lysis buffer and assayed for luciferase activity with a Dual-Luciferase Assay kit (Promega). Firefly luciferase activities were normalized based on Renilla luciferase activities. The fold induction of promoter activity was calculated by dividing the normalized luciferase activity of the stimulated cells by that of mock-treated cells. The data shown are mean values ± SD from triplicate samples.

### Immunoprecipitation

THP-1 cells were pre-incubated with HBsAg (10 µg/mL) for 24 hours before lipopolysaccharide (LPS, Invitrogen) stimulation. Cells were harvested in 1 mL of lysis buffer, 20 mM Tris-HCL (pH 7.5), 137 mM NaCl, 2 mM EDTA, 10% glycerol, 0.5% NP-40, protease inhibitors cocktail, and phosphoStop phosphatase inhibitors cocktail (Roche). The lysates were incubated overnight at 4°C with constant rotation in the presence of a rabbit polyclonal antibody against TRAF6 (Santa Cruz Biotechnology). Subsequently, protein A/G PLUS–Agarose (Santa Cruz Biotechnology) was added to the mixture and incubated at 4°C for 4 h. After washing with lysis buffer three times, the precipitates were analyzed by immunoblotting. For the detection of polyubiquitinated TRAF6 chains, the lysis buffer was supplemented with 0.5% SDS for use as a washing buffer.

### Immunoblotting

Cells were lysed in 2 × SDS Loading Buffer [100 mM Tris-HCl pH6.8, 20% (vol/vol) glycerol, 4%(wt/vol) SDS, 10% (vol/vol) β-mercaptoethanol, 0.02% (wt/vol) bromophenol blue, protease, and phosphatase inhibitors]. SDS-PAGE and western blotting were performed as previously described (9). The antibodies used were as follows: mouse anti-Flag, mouse anti-Myc, mouse antib-β-actin, mouse anti-GAPDH (Sigma), rabbit anti-TRAF6, rabbit anti-TAB2, mouse anti-Ub P4D1, rabbit anti-A20 (Santa Cruz Biotechnology), rabbit anti-phospho-JNK, rabbit anti-phospho-p38, rabbit anti- IκBα (Cell Signalling), and peroxidase-conjugated secondary goat anti-mouse and anti-rabbit antibodies (Amersham Biosciences). Protein bands were visualized using ECL Plus western blotting detection system (Perkin-Elmer) and exposed to Kodak Bio-Max film.

### Statistics

The *P* values were determined using a nonparametric one-way ANOVA and parametric unpaired or paired two-tailed T tests. The null hypothesis was rejected when *P* was < 0.05 or 0.01. *P < 0.05, **P < 0.01. Error bars in the figures represent the means with SEM.

## RESULTS

### HBsAg inhibits proinflammatory cytokines expression induced by LPS

**Fig 1:**
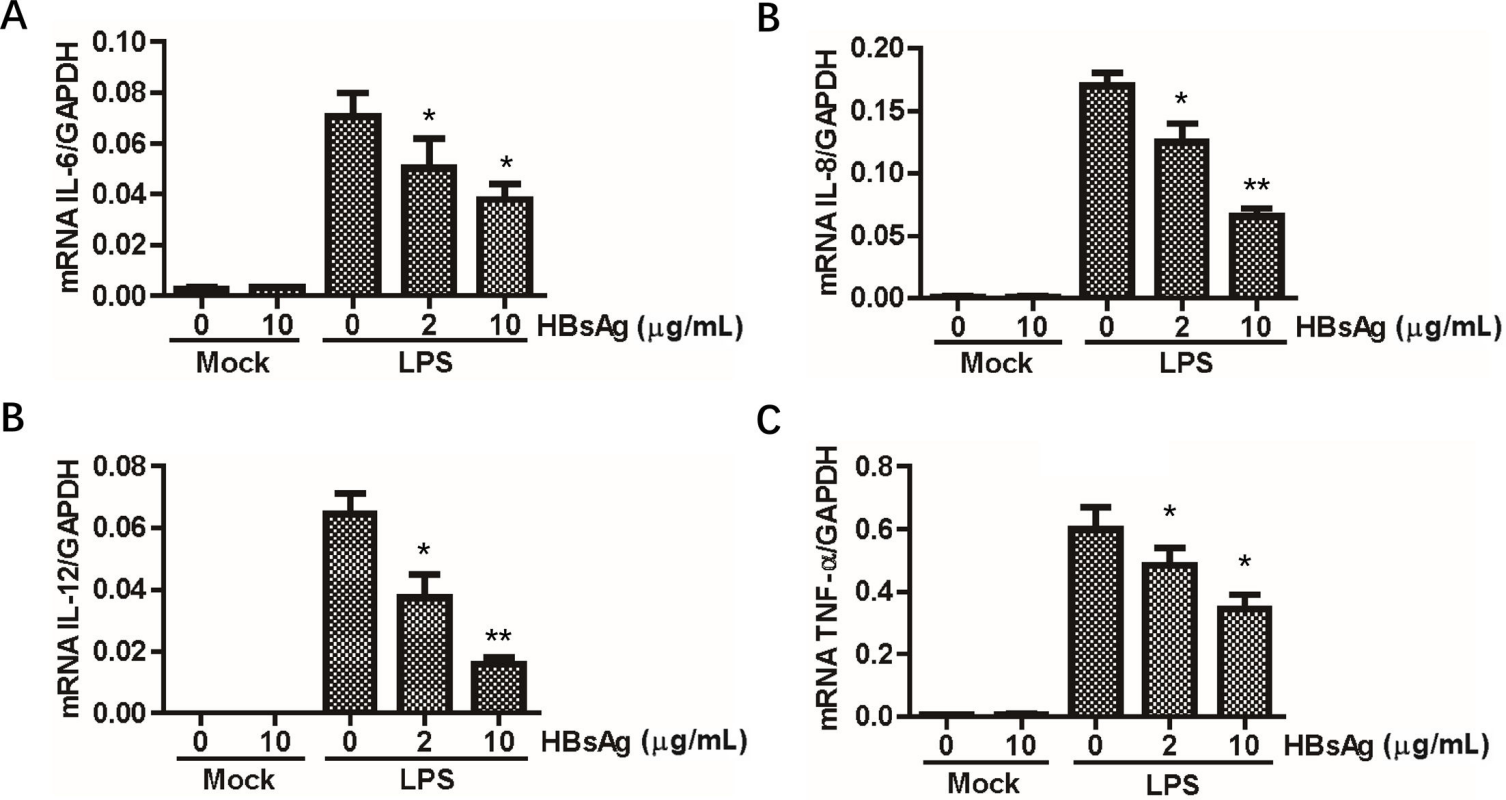
Effects of HBsAg on LPS-stimulated proinflammatory cytokines induction. THP-1 cells were pretreated with 0, 2, or 10 µg/mL HBsAg for 24 h. LPS (50 ng/mL) was added to the medium and incubated for another 6 h. Total RNA was then isolated and analyzed for IL-6 (**A**), IL-8 (**B**), IL-12 (**C**), and TNF-α (**D**) mRNA by real-time PCR. All samples were normalized using GAPDH expression. The data (mean values ± SEM) were from triplicate samples.

HBsAg might be an important factor in contributing to HBV-mediated inhibition of proinflammatory cytokines induction in PBMCs from patients with CHB as we previously described (7). To examine whether HBsAg is involved in TLR-induced cytokines transcription, the mRNA levels of IL-6, IL-8, IL-12B, and TNF-α were first examined in the two monocyte cell lines THP-1 and U937. HBsAg (0, 2, or 10 µg/mL) were added to the cell culture medium of THP-1 cells (Fig. 1A through D). Compared with the mock-treated cells, quantitative real-time polymerase chain reaction (qRT-PCR) assays showed that LPS strongly induced transcription of the cytokines. More importantly, HBsAg regulates the mRNA level of cytokines in a dose-dependent manner. Meanwhile, another monocyte cell line U937 lead to similar conclusions (Fig. S2A through D). These findings indicate that HBsAg could inhibit the immune response of monocytes induced by LPS.

### HBsAg inhibits NF-κB, JNK, and ERK activation induced by LPS

**Fig 2:**
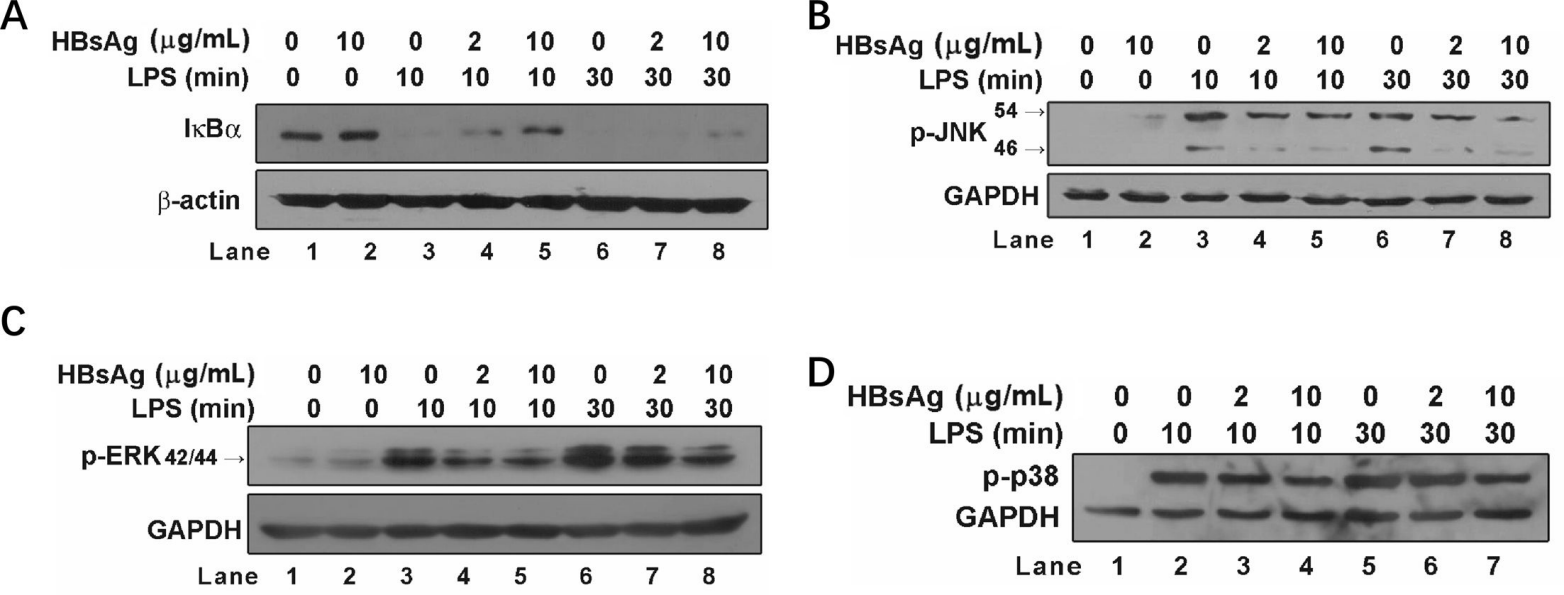
Effects of HBsAg on LPS-stimulated NF-κB and MAPKs activation. THP-1 cells were pretreated with 0, 2, or 10 µg/mL HBsAg for 24 h and stimulated with 50 ng/mL LPS for 10 or 30 min. Cells were then lysed in 2× SDS loading buffer, separated in 10% SDS-PAGE and analyzed for IκB-α (**A**), phosphorylated JNK (**B**), ERK (**C**), and p38 (**D**) by western blotting.

Because NF-κB and MAPK signaling pathways are frequently associated with TLR-mediated inflammatory responses, we next performed western blotting assays of total IκBα, phosphorylated(*P*)-JNK, p-ERK, and p-p38 to determine the regulation of signaling pathways by HBsAg. NF-κB inhibitor IκBα level was significantly increased by HBsAg pretreatment in response to LPS stimulation (Fig. 2A). Moreover, p-JNK and p-ERK levels were significantly inhibited by HBsAg pretreatment in response to LPS stimulation even at low concentrations (2 µg/mL) (Fig. 2B and C). Also, p-p38 level was inhibited by HBsAg pretreatment in response to LPS stimulation (Fig. 2D). Together, these observations suggest that HBsAg inhibits NF-κB and MAPK activation induced by LPS.

### HBsAg interfered with TLR signaling at the upstream of the cascade

**Fig 3:**
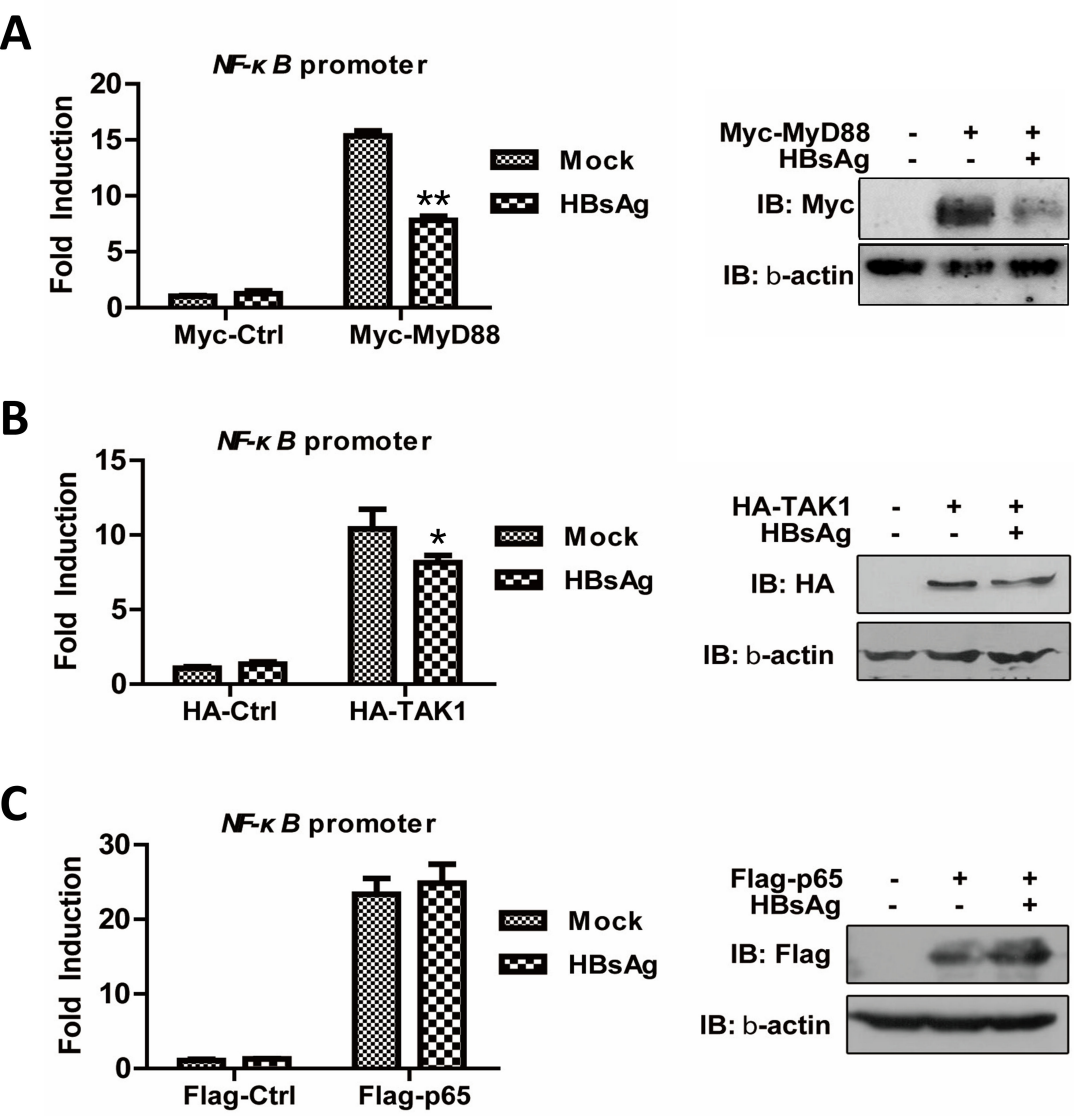
HBsAg inhibited TLR signaling upstream of TAK1 activation. THP-1 cells were co-transfected with 100 ng of pNF-κB-Luc, 25 ng of pRL-TK, and 800 ng of expression plasmids encoding MyD88 (**A**), TAK1 (**B**), or p65 (**C**). After 2 h, 10 µg/mL HBsAg was added to the medium and incubated for another 24 h. The cells were then harvested for luciferase assays (left) or for western blotting (right).

TLR signaling cascades can elicit innate immune responses that involve adaptors, kinases, and transcriptional factors. To further examine whether TLR signaling pathway was regulated by HBsAg, we performed the luciferase reporter assay by co-transfection of the NF-κB reporter with MyD88, TAK1, or p65 eukaryotic expression vectors. Compared with mock-treated cells, NF-κB activation driven by ectopic expression of MyD88 and TAK1 was inhibited in the cells treated with HBsAg (Fig. 3A and B). Meanwhile, NF-κB activation driven by p65 was not affected in the cells treated with HBsAg (Fig. 3C). Expression of the tagged proteins was determined by western blotting as shown in the right panel of Fig. 3. Collectively, these results demonstrate that TAK1 activation as early TLR signaling events was blocked by HBsAg.

### HBsAg impairs LPS-induced TRAF6-TAB2 complex formation and polyubiquitination of TRAF6

**Fig 4:**
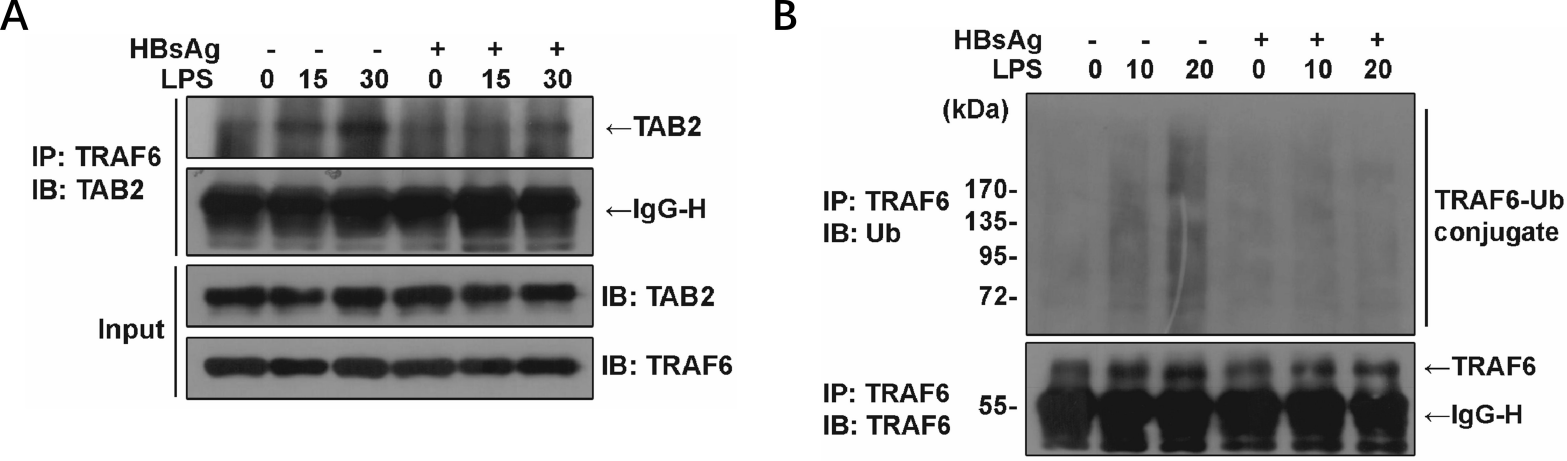
Impaired LPS-induced TRAF6-TAB2 interaction and polyubiquitination of TRAF6 in cells co-cultured with HBsAg. THP-1 cells were pre-incubated with HBsAg (10 µg/mL) for 24 h before 50 ng/mL LPS stimulation for the indicated periods of time. The cells were then lysed and analyzed by immunoprecipitation and immunoblotting. Cell lysates were subjected to co-immunoprecipitation using an anti-TRAF6 antibody, and the precipitates were analyzed by western blotting using an anti-TAB2 antibody (**A**). Cell lysates were subjected to immunoprecipitation with an antibody to TRAF6, and the precipitates were analyzed for polyubiquitination of TRAF6 by western blotting with anti-Ub antibodies (**B**).

Upon stimulation, TLR signaling cascades occur upon the formation of signaling complexes that include MyD88, IL-1 receptor-associated kinase (IRAK)−1, and TNFR-associated factor 6 (TRAF6). Among these, TRAF6 plays a central role in activating downstream signaling events. Once activated, TRAF6 undergoes autoubiquitination with K63-linked polyubiquitin chains, followed by the formation and activation of a complex composed of TGF-β-activated kinase 1 (TAK1) and its binding proteins TAB2. Activated TAK1 then leads to the activation of MAPK and NF-κB pathways, which are required for the induction of genes involved in innate immune responses. Initially, we investigated whether HBsAg could inhibit the interaction between TRAF6 and TAB2. Cell lysates treated with HBsAg or mock underwent immunoprecipitation using anti-TRAF6 antibodies, and the complexes precipitated were analyzed by western blotting. As shown in Fig. 4A, TAB2 transiently co-immunoprecipitated with TRAF6 in response to LPS stimulation in mock-treated cells but was barely detectable in TRAF6 immune complexes when HBsAg was added. Furthermore, the impact of HBsAg on LPS-induced TRAF6 polyubiquitination was examined (Fig. 4B). The results indicated that polyubiquitinated TRAF6 was exclusively detected in cells pretreated with mock, but not in those pretreated with HBsAg. These findings indicated that HBsAg impaired TLR signaling through an upstream mechanism involving defective ubiquitination of TRAF6 and assembly with TAB2.

### HBsAg upregulated TLR-negative regulator A20 expression

**Fig 5:**
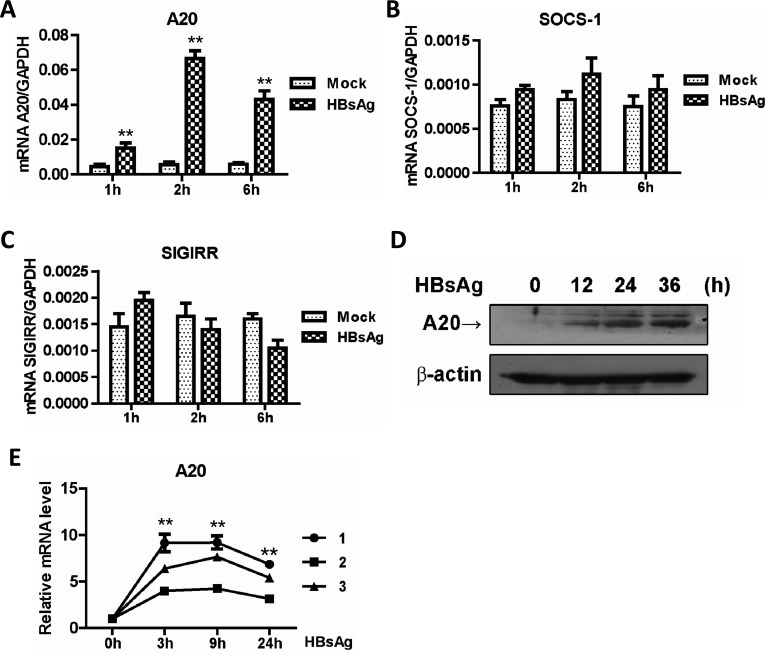
Upregulation of the TLR-negative regulator A20 expression in HBsAg-treated cells. THP-1 cells were treated with 10 µg/mL HBsAg for the indicated time periods; total RNA was isolated and analyzed for TLR-negative regulators A20 (**A**), SOCS-1 (**B**), or SIGGIR (**C**) mRNA by real-time PCR. (**D**) Protein expression levels of A20 in HBsAg-treated cells were analyzed by western blotting. (**E**) Freshly isolated PBMCs from three healthy donors were incubated with HBsAg (10 µg/mL) for the indicated periods of time. The expression level of A20 mRNA was detected by real-time PCR. The relative level of A20 mRNA was calculated by dividing the normalized mRNA values of HBsAg-treated cells by that of mock-treated cells. Paired *t* test was used.

To further clarify the mechanism of HBsAg inhibits TLR signaling, we performed a screen for established negative regulators of this pathway (10). THP-1 cells were cultured with HBsAg for 1, 2, or 6 h, and the mRNA levels of A20, SOCS-1, and SIGIRR were quantified using qPCR. Our analysis revealed that HBsAg specifically induced A20 transcription (Fig. 5A), with no significant effect on SOCS-1 and SIGIRR (Fig. 5B and C). Consistent with these findings, A20 expression was selectively upregulated in U937 cells following HBsAg treatment (Fig. S3A), whereas the expression levels of IRAKM and MyD88s remained unaltered (Fig. S3BC). To confirm the induction of A20 by HBsAg, western blotting analysis was performed using A20-specific antibodies, which confirmed increased A20 protein expression over time post-HBsAg addition (Fig. 5D**, S3D**). To extend our findings beyond cell lines, we also examined the effect of HBsAg on A20 expression in primary peripheral blood mononuclear cells (PBMCs) from healthy donors (Fig. 5E). The results showed that the mRNA levels of A20 were significantly increased upon HBsAg exposure. These results, in conjunction with the known function of A20 as a deubiquitinating enzyme capable of modifying TRAF6, suggest a potential role for A20 in the inhibition of TLR signaling by HBsAg.

### Role of A20 in HBsAg-mediated suppression of TLR signaling

**Fig 6:**
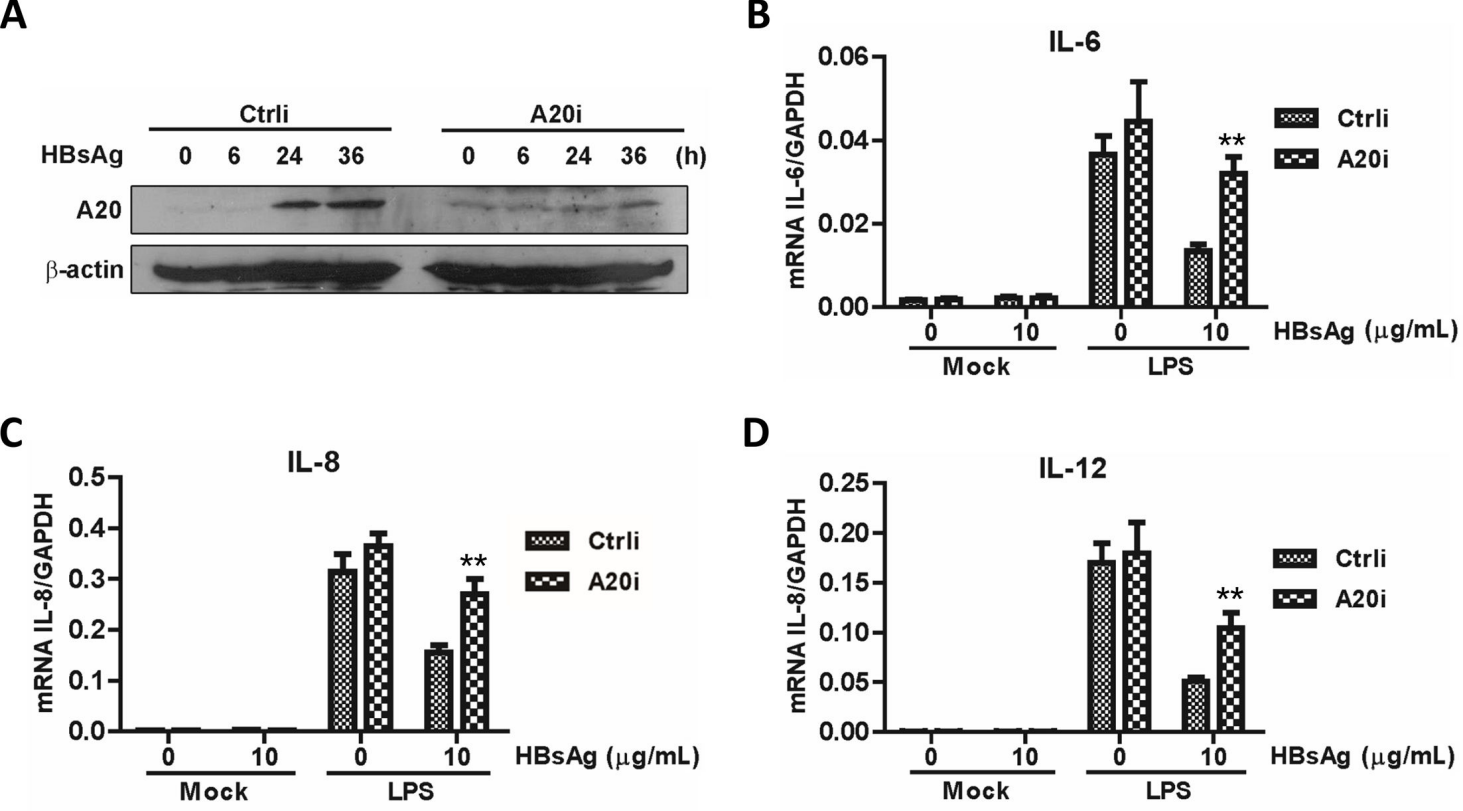
A20 is involved in the inhibition of LPS-stimulated proinflammatory cytokines induction by HBsAg. (**A**) THP-1 cells were transfected with pU6-Ctrli or pU6-A20i. In total, 10 µg/mL HBsAg was added 2 h post-transfection and incubated. Gene silencing effect was evaluated by western blotting. THP-1 cells transfected with pU6-Ctrli or pU6-A20i were treated at 10 µg/mL HBsAg for 24 h. LPS (50 ng/mL) was added to the medium and incubated for another 6 h. Total RNA was then isolated and analyzed for IL-6 (**B**), IL-8 (**C**), or IL-12 (**D**) mRNA by real-time PCR.

To examine whether A20 is involved in TLR signaling inhibition induced by HBsAg, THP-1 cells were transfected with a plasmid-encoded A20-specific siRNA (Fig. 6A). Compared with the control group, qRT-PCR assay showed that IL-6, IL-8, and IL-12 expression was significantly increased in the siRNA group (Fig. 6B–D). Therefore, in response to HBsAg, THP-1 cells increased A20 expression, which contributes to regulating TLR signaling.

## DISCUSSION

The progression of HBV infection is complicated. The interaction between the viral and host is still unknown and requires further investigation. During HBV infection, the non-infectious subviral particles of HBV, which predominantly comprise HBs, are released into the bloodstream. The concentration of particles of HBsAg may exceed 100 µg/mL in circulation. Our previous studies revealed that TLR response was attenuated in PBMCs from patients with CHB. Therefore, in response to HBV infection, the productions of IL-6, IL-8, IL-12, and TNF-α from TLR2 or TLR4 stimulation, and IFN-α from TLR9 stimulation, were inhibited, which contributed to decreased levels of plasma cytokines (7, 11). These results suggest that HBV can evade the innate immune response by interfering with the TLR2 and TLR4 systems. Then, we found that TLR2 activation was blocked by HBsAg (8), but how HBsAg impairs TLR4 cascades still needs investigation. In this study, we studied the inhibitory effects of HBsAg on TLR4 signaling transduction pathway in monocyte cell lines. We herein found that HBsAg was able to inhibit the LPS-mediated activation of MAPK and NF-κB signaling. We further demonstrated that HBsAg-inhibited LPS-mediated activation of TRAF6 contributes to resulting decreases in proinflammatory cytokines. Moreover, the expression of A20, one of the known negative regulators of TLR signaling, was specifically increased in the HBsAg-treated cells, and ablation of A20 recovered cytokines induction. It implicated that the A20 is involved in HBsAg-mediated impairment of TLR4 signaling. Collectively, the findings suggest a mechanism that may underlie the inhibition of TLR4 signaling by HBsAg, thereby impairing the immune function of monocytes.

TNF-α and IL-6 play crucial roles in controlling HBV infection (12–14), and IL-12 is known to promote antiviral immunity against various viruses, including HBV (15). Paradoxically, previous study indicates that LPS-induced TNF-α and IL-6 production is reduced in peripheral blood monocytes of CHB patients compared with those of healthy individuals (16, 17). Furthermore, HBsAg was demonstrated to impair the maturation of dendritic cells and their IL-12 production (18) and suppress the cytokines stimulated by LPS or vesicular stomatitis virus in macrophages (19). In this study, we consistently observed that monocytes pretreated with HBsAg exhibited reduced IL-6, TNF-α, and IL-12 mRNA expression following LPS stimulation. Considering that serum HBsAg levels vary significantly across different phases of chronic HBV infection, as shown by recent studies, and a decrease in HBsAg levels correlates with the induction of an effective immune response against HBV (20, 21). These findings indicate a significant immunosuppressive function of HBsAg in promoting HBV persistence through the modulation of TLR-mediated pro-inflammatory cytokine production in innate immune cells. Notably, in addition to HBsAg, CHB patients also exhibit HBeAg, which is recognized as a potent inhibitor of TLR2 signaling (22–24). Therefore, it is interesting to investigate the TLR signaling and immune cell function in CHB patients exhibiting varying serum levels of HBsAg and HBeAg, which might offer novel insights concerning the coordinated regulation of host antiviral responses by HBsAg and HBeAg.

It was reported that the expressions of COX-2, PEG2, and IL-18 were inhibited by HBsAg, due to the inactivation of NF-κB and ERK (25). However, the effect of HBsAg on the upstream components of the TLR cascade remains unclear. This study demonstrates that HBsAg inhibited both NF-κB and MAPK signaling, suggesting the presence of an upstream blockade in their activation. Given that all TLRs, with the exception of TLR3, utilize the adaptor MyD88 and a shared intracellular signaling pathway (26). We conducted a luciferase reporter assay to determine the point of HBsAg interference in TLR signaling. The results indicated that HBsAg obstructs early TLR signaling events mediated by MyD88 and TAK1, with minimal impact on downstream NF-κB signaling induced by the overexpression of p65 (Fig. 3), suggesting an upstream blockage in TLR signaling. The autoubiquitination of TRAF6 with K63-linked polyubiquitin chains and the formation of a TAK1-TAB complex are essential processes in TLR signaling that activate NF-κB and MAPK pathways, thereby inducing genes crucial for innate immune responses (27). In this study, we found that HBsAg treatment impaired the polyubiquitination of TRAF6 and the formation of the TRAF6-TAB2 complex in response to LPS stimulation (Fig. 4). Furthermore, the upregulation of A20, a ubiquitin-editing enzyme that negatively regulates TLR-initiated signaling pathways by removing ubiquitin moieties from TRAF6, was observed in this study (28, 29). The inhibitory effect of A20 on the E3 ligase activity of TRAF6, TRAF2, and cIAP1, mediated by its antagonism of the interaction between these proteins and the E2 ubiquitin-conjugating enzymes Ubc13 and UbcH5c, was observed in cells co-cultured with HBsAg (30). This mechanism is analogous to the one utilized by the measles virus *P* protein (31). However, the specific molecular mechanisms underlying HBsAg-induced upregulation of A20 are yet to be elucidated.

HBV e antigen (HBeAg) was observed to upregulate TLR4 on T cells (32) and suppress p38 phosphorylation from CD14+ cells induced by LPS (22), but to be correlated with TLR2, rather than TLR4, in the clinical cohort (33). Our previous work demonstrated a significant reduction in cell surface TLR2 expression on PBMCs from CHB patients (7), potentially leading to an overall inhibition of TLR2-mediated pro-inflammatory cytokine production. To assess whether the HBsAg-mediated suppression of LPS-induced signaling is attributed to changes in TLR4 levels, we evaluated TLR4 expression in THP-1 cells in the presence of HBsAg. A minimal decrease in surface TLR4 expression was noted (data not shown), indicating that additional, yet-to-be-determined mechanisms may contribute to the inhibition of TLR signaling by HBsAg. Alterations in A20 gene expression in PBMCs correlate with the progression of chronic hepatitis B virus infection (34). Furthermore, recent studies propose novel functions for A20, including its role as a negative regulator in RIG-I- and TLR3-mediated antiviral responses (35–38). For instance, Song et al. identified A20 as an attenuator of antigen presentation (39), and it has been implicated in the inhibition of cellular apoptosis (40). A20 mitigates pyroptosis and apoptosis in nucleus pulposus cells by promoting mitochondrial autophagy, stabilizing mitochondrial dynamics, and blocking autophagy (41, 42). The E3 ubiquitin ligase TRAF6 and the deubiquitin enzyme A20 jointly regulate ATG9A ubiquitination and autophagy activation under oxidative stress (43). Therefore, it merits investigation to determine if HBsAg modulates these critical host innate immune responses, essential for viral eradication. Additionally, hepatitis C virus, another hepatotropic virus causing chronic infections, has been reported to induce A20 expression in the human hepatic cell line HepG2 (43). Hepatitis C virus induces the ubiquitin-editing enzyme A20 via depletion of the transcription factor upstream stimulatory factor 1 to support its replication (44). Further research is needed to fully understand the impact of HBsAg on the immune regulation of both innate immune cells and hepatocytes, as well as to determine the specific direct and indirect mechanisms involved.

In conclusion, this study proposes that HBsAg disrupts TLR4 signaling and the induction of proinflammatory cytokines in monocyte cell lines through TRAF6 deubiquitination and A20 upregulation, revealing a potential mechanism of TLR4 cascade impairment by HBsAg. Subsequent investigations will validate this novel finding in clinical samples, potentially elucidating HBV evasion strategies and informing the development of new therapeutic approaches to bolster host anti-HBV immune responses.

## SUPPLEMENTAL MATERIAL

Fig. S1 spectrum.00909-24-s0001.tif Immunoblotting and silver staining of HBsAg.

Fig. S2 spectrum.00909-24-s0002.tif S2 Effects of HBsAg on LPS-stimulated proinflammatory cytokines induction.

Fig. S3 spectrum.00909-24-s0003.tif Up-regulation of the TLR-negative regulator A20 expression in HBsAg-treated cells.

Table S1 spectrum.00909-24-s0004.pdf Primer sequence used for real-time PCR.

ASM does not own the copyrights to Supplemental Material that may be linked to, or accessed through, an article. The authors have granted ASM a non-exclusive, world-wide license to publish the Supplemental Material files. Please contact the corresponding author directly for reuse.
