# Supplementary figures and images for: Hepatitis B surface antigen impairs TLR4 signaling by upregulating A20 expression in monocytes
Source: Microbiol Spectr. 2024 Sep 9;12(10):e00909-24. doi: 10.1128/spectrum.00909-24 (PMC11448406; doi:10.1128/spectrum.00909-24)

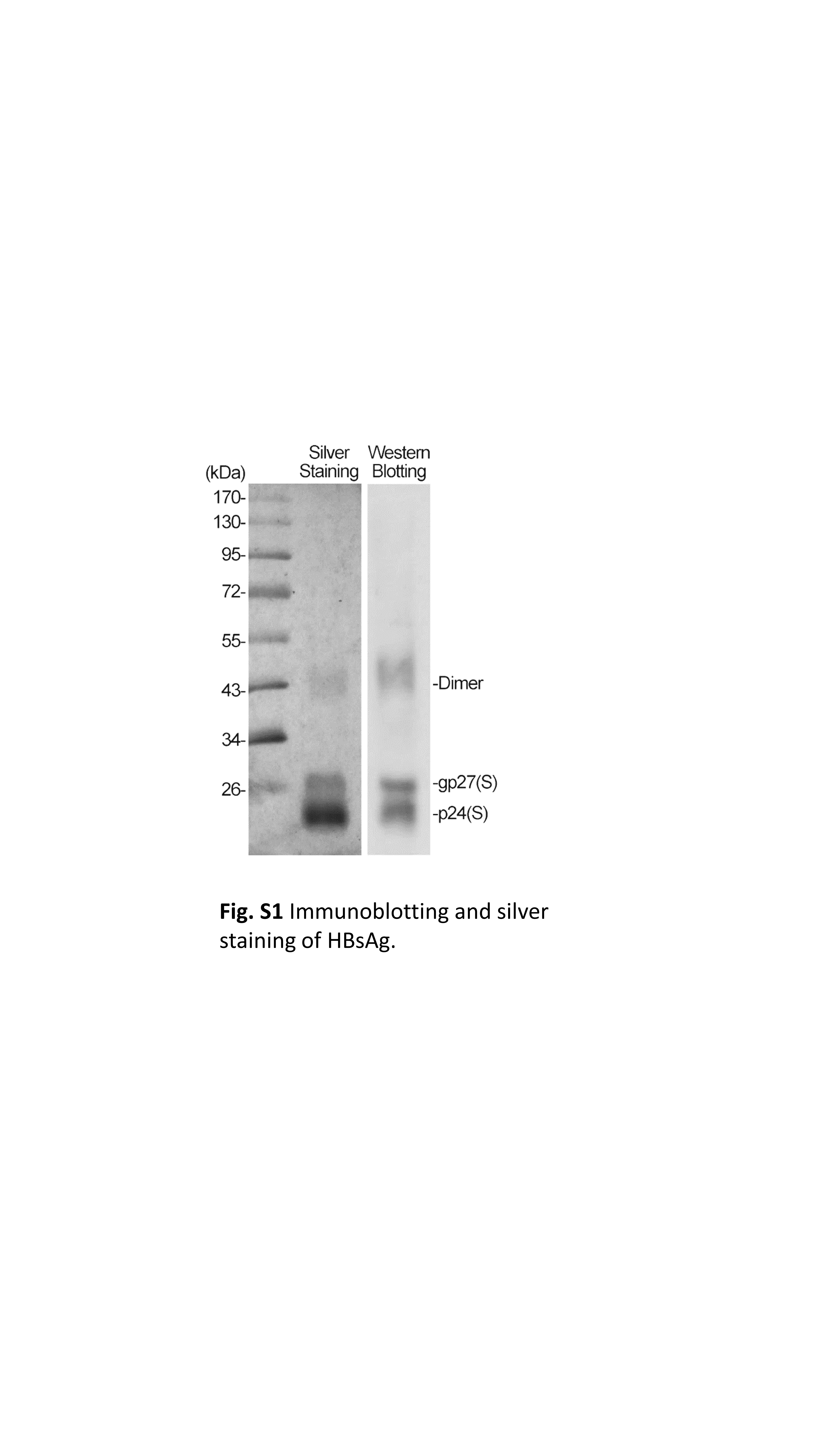

Supplement: Fig. S1 — Immunoblotting and silver staining of HBsAg. [file spectrum.00909-24-s0001.tif]

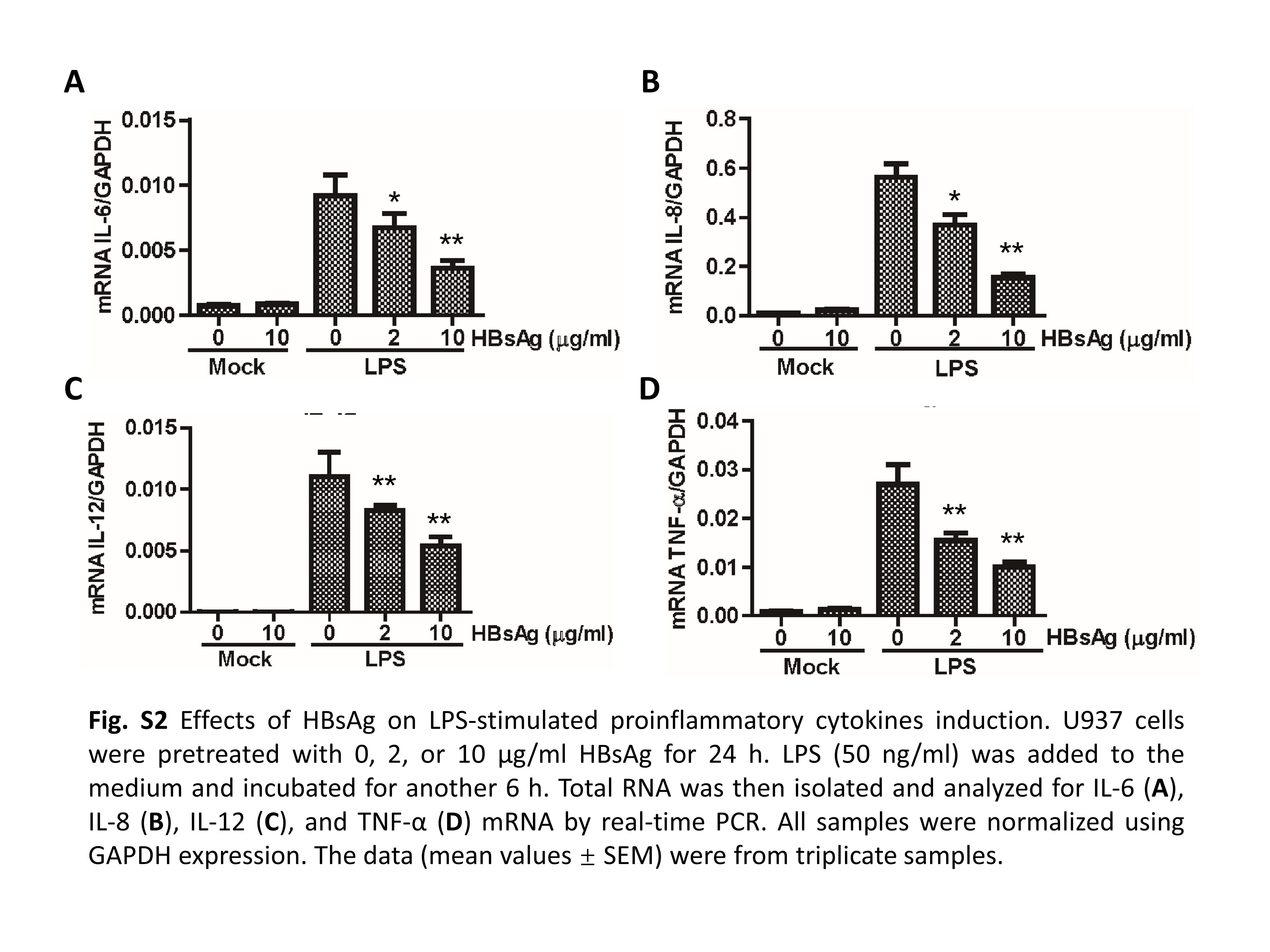

Supplement: Fig. S2 — S2 Effects of HBsAg on LPS-stimulated proinflammatory cytokines induction. [file spectrum.00909-24-s0002.tif]

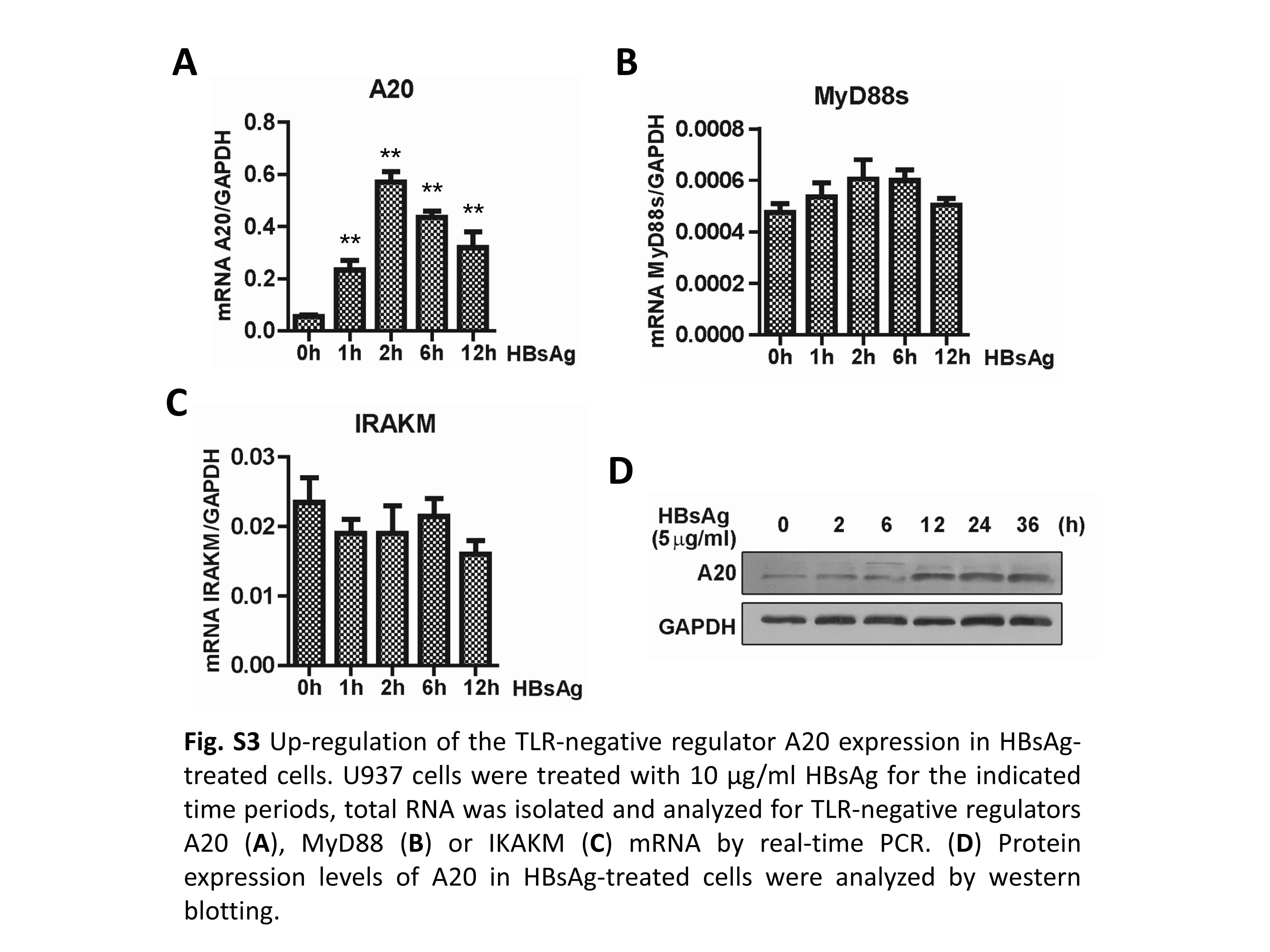

Supplement: Fig. S3 — Up-regulation of the TLR-negative regulator A20 expression in HBsAg-treated cells. [file spectrum.00909-24-s0003.tif]
