# Supplementary material for: Hepatitis B surface antigen impairs TLR4 signaling by upregulating A20 expression in monocytes
Source: Microbiol Spectr. 2024 Sep 9;12(10):e00909-24. doi: 10.1128/spectrum.00909-24 (PMC11448406; doi:10.1128/spectrum.00909-24)
Supplement: Table S1 — Primer sequence used for real-time PCR. [file spectrum.00909-24-s0004.pdf]

Table S1 Primer sequence used for real-time PCR

Gene	Primer Sequences (5'→3')	Length
IL-6	F- CCCAGTACCCCCAGGAGAAGATTC<br>R- GCCAGTGCCTCTTTGCTGCTT	174 bp
IL-8	F- GAAGGTGCAGTTTTGCCAAGGA<br>R- CACCCAGTTTTCCTTGGGGTC	195 bp
IL-12p40	F- TGGAGTGCCAGGAGGACAGTG<br>R- AGTTCTTGGGTGGGTCAGGTTTG	150 bp
TNF-α	F- GTTCCCCAGGGACCTCTCTCTAAT<br>R- TCTCAGCTCCACGCCATTGG	174 bp
A20	F- CAAAATGGCTTCCACAGACACACCC<br>R- GGAGAGGCAAGTAAATTCCACCCA	194 bp
IRAKM	F- AAGTATGTAGACCAAGGTAAAAGT<br>R- CTGATAACTCTTCTCTGAAGG	174 bp
MyD88s	F- TGGGACCCAGCATTGGGCAT<br>R- AGACCAGACACAGGTGCCAGG	170 bp
SOCS-1	F- CGACACGCACTTCCGCACATTC<br>R- AAAAGCAGTTCCGCTGGCGG	185 bp
SIGIRR	F- GGGTCAACGTGACCAGCACTGAAG<br>R- GCAGCACGTTGAGACGGCACTT	192 bp
GAPDH	F- GGTATCGTGGAAGGACTCATGA<br>R- ATGCCAGTGAGCTTCCCGTTCAGC	188 bp
